# Influence of coronary artery disease and percutaneous coronary intervention on mid‐term outcomes in patients with aortic valve stenosis treated with transcatheter aortic valve implantation

**DOI:** 10.1002/clc.23655

**Published:** 2021-05-25

**Authors:** Toshiki Kaihara, Takumi Higuma, Masaki Izumo, Nozomi Kotoku, Tomomi Suzuki, Haruka Kameshima, Yukio Sato, Shingo Kuwata, Masashi Koga, Takanobu Mitarai, Mika Watanabe, Kazuaki Okuyama, Ryo Kamijima, Yuki Ishibashi, Kihei Yoneyama, Yasuhiro Tanabe, Tomoo Harada, Yoshihiro J. Akashi

**Affiliations:** ^1^ Division of Cardiology, Department of Internal Medicine St. Marianna University School of Medicine Kawasaki Japan; ^2^ Department of Cardiology St. Marianna University School of Medicine, Toyoko Hospital Kawasaki Japan

**Keywords:** coronary artery disease, severe aortic valve stenosis, transcatheter aortic valve implantation

## Abstract

**Background:**

A high frequency of coronary artery disease (CAD) is reported in patients with severe aortic valve stenosis (AS) who undergo transcatheter aortic valve implantation (TAVI). However, the optimal management of CAD in these patients remains unknown.

**Hypothesis:**

We hypothesis that AS patients with TAVI complicated by CAD have poor prognosis. His study evaluates the prognoses of patients with CAD and severe AS after TAVI.

**Methods:**

We divided 186 patients with severe AS undergoing TAVI into three groups: those with CAD involving the left main coronary (LM) or proximal left anterior descending artery (LAD) lesion (the CAD[LADp] group), those with CAD not involving the LM or a LAD proximal lesion (the CAD[non‐LADp] group), and those without CAD (Non‐CAD group). Clinical outcomes were compared among the three groups.

**Results:**

The CAD[LADp] group showed a higher incidence of major adverse cardiovascular and cerebrovascular events (MACCEs) and all‐cause mortality than the other two groups (log‐rank p = .001 and p = .008, respectively). Even after adjustment for STS score and percutaneous coronary intervention (PCI) before TAVI, CAD[LADp] remained associated with MACCE and all‐cause mortality. However, PCI for an LM or LAD proximal lesion pre‐TAVI did not reduce the risk of these outcomes.

**Conclusions:**

CAD with an LM or LAD proximal lesion is a strong independent predictor of mid‐term MACCEs and all‐cause mortality in patients with severe AS treated with TAVI. PCI before TAVI did not influence the outcomes.

## 1 INTRODUCTION

Moderate or severe mitral and aortic valvular diseases are extremely common in elderly populations, and their incidence increases with age.^1^ In Japan and worldwide, the population is aging rapidly and the problems presented by valvular diseases have become a serious public health issue. Transcatheter aortic valve implantation (TAVI) is one of the treatment options for severe aortic valve stenosis (AS), especially for older patients. In 2019, the Placement of AoRTic traNscathetER valves (PARTNER) 3 trial^2^ and the Evolut Low Risk Trial^3^ revealed the effectiveness of TAVI for even low‐risk patients. It is expected that the number of TAVI cases will continue to grow. In severe AS patients, the reported prevalence of coronary artery disease (CAD) as a comorbid disorder has ranged widely from 11%^4^ to 63%.^5^ Among the previous studies, the PARTNER trials, which are worldwide randomized clinical trials of TAVI, reported a comparably higher rate of concomitant CAD with severe AS.^6^, ^7^ However, the optimal management method for patients with both CAD and severe AS has not been established. In the era of TAVI, it is important to determine whether TAVI can and should be performed before percutaneous coronary intervention (PCI) for elderly patients with CAD and severe AS. It is not yet known whether or not concomitant CAD in severe AS patients increases the risks posed by the TAVI procedure, and fewer data have been published from relevant studies in Asia compared to those from Europe and the U.S. Left main coronary (LM) artery CAD and proximal left anterior descending (LAD) artery CAD are considered high‐risk features because these arteries supply the large area of myocardium. However, the strategy of conducting a PCI before TAVI for coronary lesions has not been established. We therefore retrospectively analyzed the prognoses of patients with CAD and severe AS who underwent TAVI.

## 2 METHODS

### 2.1 Study population and study design

**FIGURE 1:**
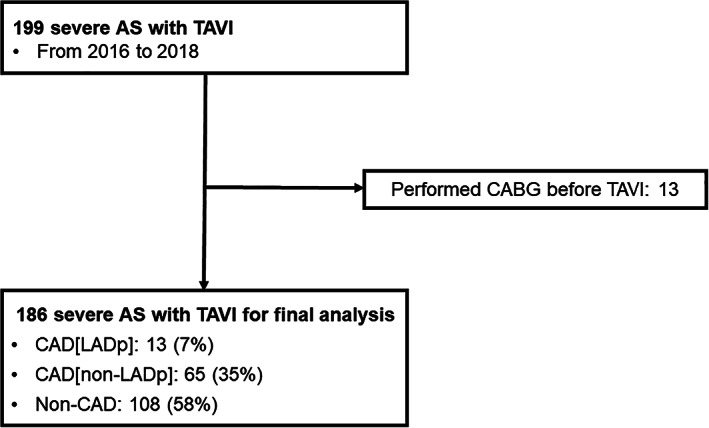
Flowchart of this study. AS, aortic stenosis; CABG, coronary artery bypass grafting; CAD, coronary artery disease, LAD, left anterior descending artery; CAG, coronary angiography; PCI, percutaneous coronary intervention; TAVI, transcatheter aortic valve implantation

This study was a retrospective single‐center cohort study. We analyzed the cases of the series of 199 consecutive symptomatic patients with severe AS who were treated with TAVI at St. Marianna University School of Medicine, Kawasaki, Japan since January 2016. All patients' cases and options were discussed by our cardiology team for appropriate adaptation. Before undergoing the TAVI, the patients were examined by coronary angiography (CAG) or coronary computed tomography angiography (CTA). We excluded 13 patients who underwent coronary artery bypass grafting (CABG) (Figure 1).

This study was a retrospective observational study. It was approved by St. Marianna University School of Medicine Ethics Committee (ID: 5078). Informed consent was acquired in the form of opt‐out on the website.

We divided the 186 remaining patients into the following three groups. The CAD[LADp] group (*n* = 13) was comprised of the patients in whom CAD (as defined below) was detected in the LM or proximal LAD (five and six segments in American Heart Association [AHA] classification) by CAG or CTA. The CAD[non‐LADp] group (*n* = 65) was the patients in whom CAD was detected at sites other than the LM and proximal LAD by CAG or CTA. The non‐CAD group (*n* = 108) was the patients in whom CAD was not detected by CAG or CTA. CAD was defined as >75% stenosis of ≥1 major branch on CAG/CTA or 50% stenosis only in the LM based on the AHA classification derived from the visual assessment of CAG or CTA results. The angiographic assessment of CAD was based on the consensus of three experienced cardiologists. If PCI had been performed previously and there was no re‐stenosis at the time of coronary screening, the patient was assigned to the non‐CAD group. When deciding whether to perform PCI for the patients with CAD, we took into consideration the ischemic burden, the technical complexity, the risk of in‐stent restenosis, and the patient's general condition (e.g., the presence of chronic kidney disease). PCI for CAD detected by this coronary screening was basically implemented before TAVI as an independent hospitalization.

After discharge, the outpatient follow‐up examinations were performed at 1 month, 12 months, and then annually. We conducted a follow‐up survey of the patients' outcomes by checking their clinical records and consulting the patients' referring physicians.

### 2.2 TAVI and PCI procedures

Before each TAVI procedure, we generally added on antiplatelet therapy (100 mg of aspirin or 75 mg of clopidogrel) or anticoagulant therapy (warfarin or direct oral anticoagulants). Procedural anticoagulation was achieved by heparinization, and the target activated clotting time was 250–300 msec. We used two TAVI systems: the Medtronic CoreValve™ or Evolut R™/PRO™ (Medtronic, Minneapolis, MN) and the Edwards SAPIEN XT™/3™ (Edwards Lifesciences, Irvine, CA). The choice of valve type was discussed by a heart team conference made up of cardiologists, cardiac surgeons, anesthesiologists, nurses, and clinical engineers. Balloon expandable valves were generally chosen for CAD patients indicated for PCI, so that the coronary artery could be easily accessed after TAVI. However, consideration was also made for factors other than the coronary artery itself (e.g., the status of the access route, the annulus size, patient frailty, the presence or absence of atrioventricular block) in order to make a comprehensive decision. All patients were initially screened for a transfemoral approach. In cases in which transfemoral access was not possible, the patients were evaluated for alternative access sites in the following order of preference: subclavian arteries, left ventricular apex, ascending aorta.

The PCI procedure was initiated following the administration of unfractionated heparin (100 IU/kg) into the peripheral vein. The decision to choose either a drug‐eluting stent or a drug‐coated balloon was left to the operators' discretion. The patients generally continued dual antiplatelet therapy for 1 year after the PCI.

### 2.3 Outcomes

The primary outcome was the major adverse cardiovascular and cerebrovascular events (MACCEs) defined as non‐lethal myocardial infarction, unstable angina pectoris, heart failure requiring hospitalization, stroke, and cardiovascular death. The secondary outcome was all‐cause mortality.

### 2.4 Statistical analyses

All statistical analyses were carried out with SPSS/Windows, ver. 22.0 (SPSS, Chicago, IL). Data are presented as the mean (±*SD*) or as frequencies (percentages). We used the chi‐squared test, an analysis of variance, and the Kruskal‐Wallis test to examine differences among three groups, and we performed a Kaplan–Meier analysis and a multivariate Cox regression analysis to examine changes over time to the endpoints. Bonferroni corrected post‐hoc tests were conducted to determine where these differences occurred. A two‐tailed p value <.05 was considered significant.

## 3 RESULTS

A total of 186 consecutive patients underwent a TAVI, with a follow‐up duration of 405 ± 236 days. The baseline characteristics of the patients of three groups in this study are summarized in Table 1. Among the 186 patients, the mean age was 84 years old; 85% were hypertensive; 50% had dyslipidemia; 26% had diabetes. The 8% had a past medical history of angina pectoris; 14% had peripheral artery disease; and 12% had cerebrovascular disease. The prevalence of CAD in the patients was 42%. PCI was successfully performed without any complications in 29 patients.

**TABLE 1:** The baseline characteristics

			Group			p	Bonferroni corrected p		
			CAD[LADp] (*n* = 13)	CAD[non‐LADp] (*n* = 65)	Non‐CAD (*n* = 108)		CAD[LADp] vs. CAD[non‐LADp]	CAD[non‐LADp] vs. Non‐CAD	CAD[LADp] vs. Non‐CAD
Age, y			83 ± 3	84 ± 6	84 ± 6	0.61			
Male sex, *n* (%)			3 (23)	28 (43)	28 (26)	.050			
Current smoking, *n* (%)			0 (0)	5 (8)	3 (3)	0.22			
Past medical history	Diabetes, *n* (%)		5 (38)	21 (32)	22 (20)	0.12			
	Dyslipidemia, *n* (%)		8 (62)	35 (54)	50 (46)	0.43			
	Hypertension, *n* (%)		11 (85)	60 (92)	87 (81)	.088			
	Angina pectoris, *n* (%)		2 (15)	11 (17)	1 (1)	<.001	0.89	<0.001	0.002
	Peripheral artery disease, *n* (%)		6 (46)	5 (8)	15 (14)	.001	<0.001	0.22	0.004
	Cerebrovascular disease, *n* (%)		1 (8)	8 (12)	14 (13)	0.86			
CAD	Single vessel, *n* (%)		5 (38)	40 (60)	N/A	0.15			
	Multi vessels, *n* (%)		8 (62)	25 (40)					
Syntax score^a^			11 (9–20)	9 (5–11)	N/A	.006			
PCI	not performed, *n* (%)		6 (46)	43 (66)	N/A	<.001			
	involving LM or LAD proximal lesion, *n* (%)		7 (54)	0 (0)					
	not involving LM nor LAD proximal lesion, *n* (%)		0 (0)	22 (34)					
Hemoglobin, g/dl			10.8 ± 1.1	11.0 ± 1.9	11.2 ± 1.5	0.73			
Creatinine, mg/dl			1.1 (0.8–1.5)	0.9 (0.7–1.2)	0.9 (0.7–1.2)	0.27			
C reactive protein, mg/dl			0.07 (0.02–0.45)	0.10 (0.05–0.29)	0.07 (0.02–0.40)	0.62			
LDL cholesterol, mg/dl			109 ± 40	97 ± 28	100 ± 24	0.34			
HDL cholesterol, mg/dl			49 ± 11	54 ± 13	58 ± 18	.064			
Triglyceride, mg/dl			121 (66–159)	84 (74–123)	90 (69–117)	0.68			
NT‐proBNP^b^, pg/ml			1302 (926–5307)	1032 (517–2303)	1035 (500–2462)	0.26			
STS score			7.7 (4.8–11.7)	5.6 (3.8–8.2)	4.8 (3.6–7.0)	.020	0.080	0.11	0.011
Logistic euroSCORE			31.1 (14.4–39.6)	20.3 (13.6–32.5)	10.1 (7.9–15.2)	<.001	0.14	<0.001	<0.001
LVEF, %			71 (59–75)	66 (59–71)	65 (58–71)	0.36			
Annulus area analyzed by CT, mm^2^			403 (366–436)	403 (347–464)	382 (338–436)	0.47			
TAVI Procedure time, min			143 (86–182)	107 (76–143)	100 (76–125)	0.13			
Valve type	SAPIEN™ series, *n* (%)		12 (92)	57 (88)	92 (85)	0.74			
	CoreValve™ or Evolut™ series, *n* (%)		1 (8)	8 (12)	16 (15)				
Valve size	SAPIEN™ series	20 mm, *n* (%)	0 (0)	6 (9)	10 (9)	0.70			
		23 mm, *n* (%)	8 (62)	33 (51)	55 (51)				
		26 mm, *n* (%)	3 (23)	16 (25)	23 (21)				
		29 mm, *n* (%)	1 (8)	2 (3)	4 (4)				
	CoreValve™ or Evolut™ series	26 mm, *n* (%)	1 (8)	2 (3)	11 (10)				
		29 mm, *n* (%)	0 (0)	6 (9)	5 (5)				
On‐admission	RAS^**c**^ inhibitors, *n* (%)		4 (31)	32 (49)	44 (41)	0.36			
	Beta‐blockers, *n* (%)		10 (77)	25 (38)	40 (37)	.020	0.011	0.85	0.006
	Antiplatelet agents, *n* (%)		11 (85)	56 (86)	74 (69)	.024	0.88	0.009	0.23
On‐discharge	RAS inhibitors, *n* (%)		5 (38)	38 (58)	45 (42)	.081			
	Beta‐blockers, *n* (%)		10 (77)	25 (38)	40 (37)	.022	0.020	0.56	0.006
	Antiplatelet agents, *n* (%)		11 (85)	56 (86)	77 (71)	.008	0.50	0.002	0.31
Pre‐TAVI TTE	AVA, cm^2^		0.6 (0.4–0.8)	0.6 (0.5–0.7)	0.6 (0.5–0.7)	0.98			
	AVPV, m/s		3.6 (3.3–4.6)	4.5 (3.7–5.1)	4.2 (3.6–4.9)	0.21			
	Mean AVPG, mmHg		29 (25–52)	44 (30–58)	41 (29–54)	0.26			
Post‐TAVI TTE	EOA, cm^2^		1.6 (1.3–1.9)	1.4 (1.2–1.7)	1.4 (1.1–1.6)	0.24			
	AVPV, m/s		1.9 (1.6–2.3)	2.1 (1.8–2.4)	2.3 (1.8–2.6)	.077			
	Mean AVPG, mmHg		7 (6–11)	9 (7–12)	10 (7–14)	.062			

Abbreviations: AVA, aortic valve area; AVPV, aortic valve peak velocity; AVPG, aortic valve pressure gradient; EOA, effective orifice area; CAD, coronary artery diseases; LM, left main coronary artery; RCA, right coronary artery; LAD, left anterior ascending artery; LCX, left circumflex artery; STS, Society of Thoracic Surgeons; LVEF, left ventricular ejection fraction; TAVI, transcatheter aortic valve implantation; TTE, transthoracic echocardiography.

*Note*: Values are expressed as the mean ± *SD* or the median (Q1–Q3) or number (percentage) unless otherwise indicated. Bonferroni correction in which p < .0167 is significant.

^**a**^ Syntax score was calculated only for patients performed coronary angiography.

^**b**^ N‐terminal pro‐brain natriuretic peptide (NT‐proBNP) was adjusted by 6th the level of BNP in patients who did not have NT‐proBNP data.^19^

^**c**^ Renin‐angiotensin system (RAS) inhibitors include either angiotensin‐converting enzyme inhibitors or angiotensin II receptor blockers.

The non‐CAD group had significantly lower STS scores and logistic euroSCOREs compared to the CAD[LADp] group (4.8 vs. 7.7, p = 0.011; 10.1 vs. 31.1, p < .001, respectively). The CAD[LADp] and CAD[non‐LADp] groups had significantly higher rates of a history of angina pectoris compared to the non‐CAD group (15% vs. 1%, p = .002; 17% vs. 1%, p < .001, respectively). The CAD[non‐LADp] group had a significantly higher rate of antiplatelet drug use on admission and at discharge than the non‐CAD group (86% vs. 69%, p = .009; 86% vs. 71%, p = .002, respectively). The CAD[LADp] group had significantly higher SYNTAX scores than the CAD[non‐LADp] group (11 vs. 9, p = .006).

**FIGURE 2:**
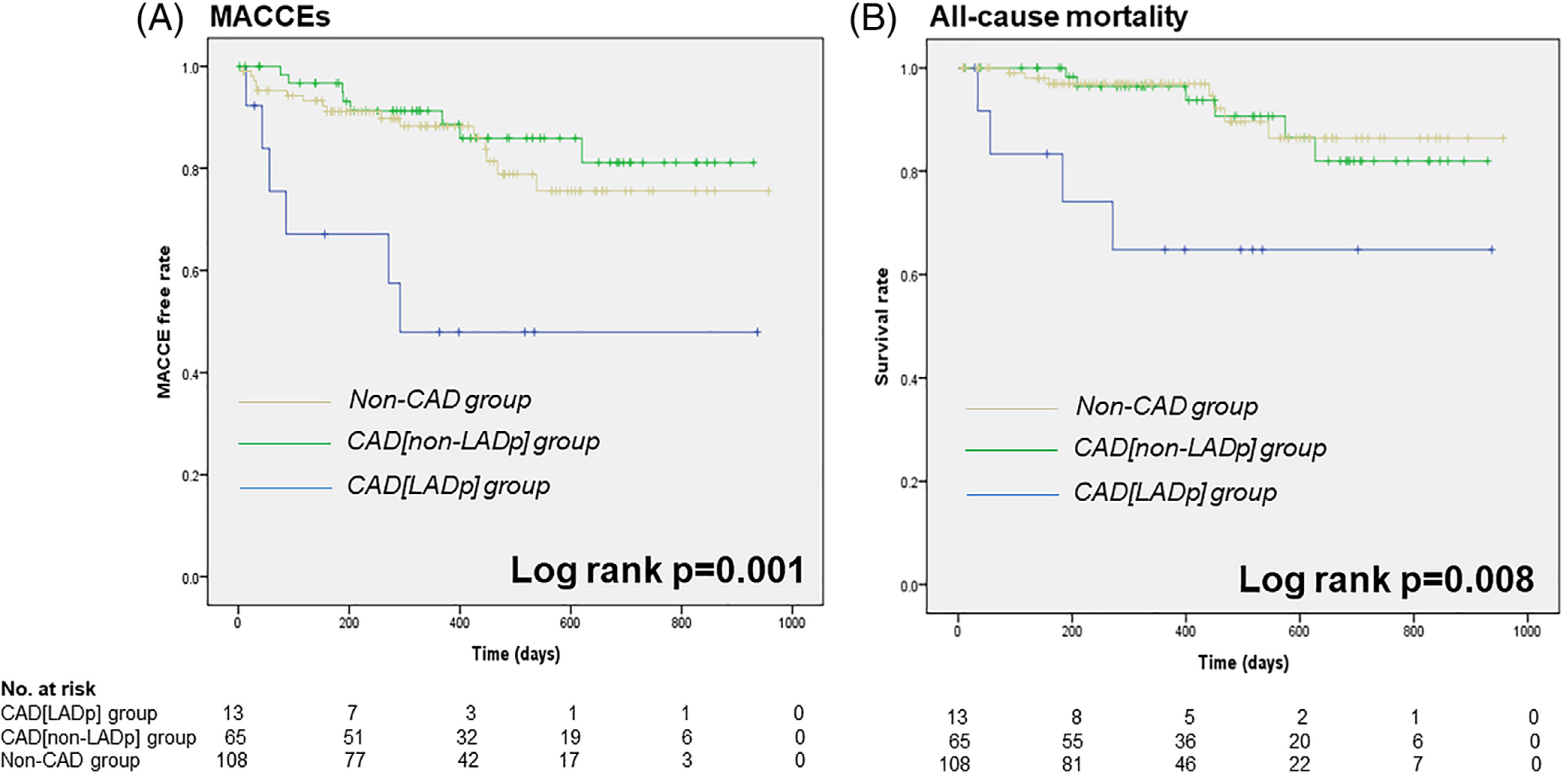
Kaplan–Meier Curves for (A) MACCEs and (B) all‐cause mortality for the patients in the CAD[LADp] group (*blue*), those in the CAD[non‐LADp] group (*green*), and those in the non‐CAD group (*yellow*). MACCEs, major adverse cardiovascular and cerebrovascular events

During the follow‐up period, the total number of MACCEs was 30 and the total number of deaths was 17 (Table 2). The results of the Kaplan–Meier analysis are displayed in Figure 2. The CAD[LADp] group showed significantly higher incidences of MACCEs and all‐cause mortality compared to the other two groups (log rank p = .001 for MACCEs; log‐rank p = .008 for all‐cause mortality).

**TABLE 2:** MACCEs and all‐cause mortality of patients in each group

		Group		
		CAD[LADp]	CAD[non‐LADp]	Non‐CAD
MACCEs	Myocardial infarction, *n* (%)	3 (30)	1 (7)	0 (0)
	Heart failure, *n* (%)	1 (10)	5 (36)	10 (43)
	Stroke, *n* (%)	0 (0)	0 (0)	2 (9)
	Cardiovascular death, *n* (%)	2 (20)	2 (14)	4 (17)
All‐cause mortality	Cardiovascular death, *n* (%)	3 (30)	4 (29)	6 (26)
	Non‐cardiovascular death, *n* (%)	1 (10)	2 (14)	1 (4)

Abbreviations: CAD, coronary artery disease; LAD, left anterior descending artery; MACCEs, major adverse cardiac or cerebrovascular events.

The univariate analysis demonstrated that CAD in an LM or LAD proximal lesion was significantly associated with increased incidences of MACCEs (hazard ratio [HR] 4.01, 95% confidence interval [CI]: 1.57–10.28) and an increased incidence of all‐cause mortality (HR 5.32, 95% CI: 1.55–18.21). Even after adjustment for the STS score and the performance of a PCI before TAVI, CAD involving an LM or LAD proximal lesion remained associated with increased incidences of MACCEs (HR 3.67, 95% CI: 1.07–12.61) and all‐cause mortality (HR 5.21, 95% CI: 1.08–25.21). PCI involving an LM or LAD proximal lesion before TAVI did not have a significant impact on MACCEs (HR 1.13, 95% CI: 0.22–5.92) or all‐cause mortality (HR, 1.03; 95% CI: 0.14–7.42) (Table 3). The results of the Kaplan–Meier analysis revealed that performing a PCI for an LM or LAD proximal lesion did not have a significant influence on MACCEs or all‐cause mortality in the CAD[LADp] group, either (Figure S1).

**TABLE 3:** Multivariate Cox regression analysis for each outcome

A. MACCEs						
	Crude model		Model 1		Model 2	
	HR (95% CI)	p	HR (95% CI)	p	HR (95% CI)	p
Non‐CAD	Ref.		Ref.		Ref.	
CAD involving LM or LAD proximal lesion	4.01 (1.57–10.28)	.004	3.87 (1.49–10.04)	.005	3.67 (1.07–12.61)	.039
CAD not involving LM nor LAD proximal lesion	0.72 (0.31–1.68)	0.45	0.71 (0.30–1.66)	0.43	0.92 (0.38–2.25)	0.85
High STS score (≥8)			1.21 (0.55–2.68)	0.63	1.16 (0.51–2.64)	0.72
Non‐PCI					Ref.	
PCI involving LM or LAD proximal lesion					1.13 (0.22–5.92)	0.88
PCI not involving LM nor LAD proximal lesion					0.30 (0.037–2.47)	0.27

B. All‐cause mortality						
	Crude model		Model 1		Model 2	
	HR (95% CI)	p	HR (95% CI)	p	HR (95% CI)	p
Non‐CAD	Ref.		Ref.		Ref.	
CAD involving LM or LAD proximal lesion	5.32 (1.55–18.21)	.008	5.28 (1.53–18.14)	.008	5.21 (1.08–25.21)	.040
CAD not involving LM nor LAD proximal lesion	1.17 (0.39–3.48)	0.78	1.16 (0.39–3.47)	0.79	1.35 (0.42–4.29)	0.61
High STS score (≥8)			1.09 (0.38–3.12)	0.87	1.06 (0.37–3.06)	0.91
Non‐PCI					Ref.	
PCI involving LM or LAD proximal lesion					1.03 (0.14–7.42)	0.98
PCI not involving LM nor LAD proximal lesion					0.51 (0.59–4.42)	0.54

*Note*: Model 1: adjusted for high STS score (≥8). Model 2: adjusted for Model 1 and PCI for LAD proximal lesion / non‐LAD proximal lesion before TAVI.

Abbreviations: CI, confidence interval; CAD, coronary artery disease; LAD, left anterior descending artery; MACCEs, major adverse cardiac or cerebrovascular events; HR, hazard ratio.

## 4 DISCUSSION

The main findings of this study were as follows. (1) The prevalence of CAD in patients with severe AS treated by TAVI was 42%. (2) The CAD[LADp] group had a higher incidence of MACCE and a higher incidence of all‐cause mortality than the other two patient groups. (3) The multivariate Cox regression adjusted for the STS score and the performance of a PCI before TAVI revealed that CAD involving an LM or LAD lesion was independently associated with increased rates of MACCEs and all‐cause mortality. (4) PCI before TAVI did not affect the patients' outcomes.

In this series of patients with severe AS, the prevalence of CAD was 42%. In previous TAVI studies the prevalences of CAD ranged from 11%^4^ to 63%,^5^ and our present results were roughly intermediate between these findings.

The impact of coexisting CAD on clinical outcomes in TAVI patients is very controversial. Dewey et al. reported that coexisting CAD negatively influenced procedural outcomes and long‐term survival in TAVI patients.^8^ On the other hand, Ussia et al. reported that CAD coexisting with severe AS did not impact procedural outcomes and mid‐term incidence of MACCE and survival in elderly patients undergoing TAVI.^9^ However, a multivariate analysis performed in a large number of TAVI patients in the UK reported that CAD was one of the independent predictors of 2‐year mortality.^10^ We classified the present TAVI candidates into three groups, and our analyses revealed that (1) the CAD[LADp] group had higher incidences of MACCEs and all‐cause mortality than the other two groups, and (2) CAD involving an LM or LAD proximal lesion was an independent prognostic factor for MACCEs and all‐cause mortality. Our present study indicates that CAD influences the prognosis of those TAVI candidates with lesions in the LM or proximal LAD, which supply the largest area of myocardium among the coronary arteries.

We also found that the performance of a PCI before the TAVI did not influence the incidence of MACCEs or all‐cause mortality in the multivariate Cox regression analysis. A previous report showed that there was no difference in 2‐year mortality between PCI plus TAVI and only TAVI.^11^ A greater magnitude of improvement of coronary hemodynamics can be obtained by TAVI than by PCI. Furthermore, it has been reported that PCI without an appropriate assessment of myocardial ischemic burden does not improve clinical outcomes in patients with stable angina pectoris. The Clinical Outcomes Utilizing Revascularization and Aggressive Drug Evaluation (COURAGE) trial showed that an addition of PCI to optimal medical therapy (OMT) did not reduce the risk of death, myocardial infarction, or other major cardiovascular events in patients with stable angina pectoris,^12^ which may support our present findings. Another study demonstrated that performing a PCI along with a TAVI during the patient's same hospital admission is associated with a higher mortality compared to performing TAVI alone.^13^ Unlike the results of that investigation, our present study did not find that PCI is harmful. This difference may be attributable to the fact that the timing of the PCI, the coronary artery anatomy, and the SYNTAX score were more thoroughly analyzed in our present investigation At least two studies have described that coronary revascularization before TAVI seemed to be safe, and the outcomes of this strategy were the same as those observed in non‐CAD TAVI patients.^14^, ^15^ Our present findings also suggest that coronary revascularization before TAVI is safe, as supported by other studies.

On the other hand, it is reported that the reduction in ischemic burden achieved by PCI reduces the risk for death or myocardial infarction. A COURAGE trial nuclear substudy suggested that a treatment target of more than 5% ischemia reduction is suitable for adding PCI to OMT.^16^ However, this finding is not conclusive since the results obtained using the current method of measuring the ischemic burden in severe AS patients are inconsistent. Fractional flow reserve (FFR) guided PCI may change the prognosis,^17^ but it can be challenging to define the optimal timing to assess the functional severity of coronary artery lesions using the flow wire method in patients with AS. A recent report showed that coronary hemodynamics are influenced by TAVI and AS removal.^18^ In addition, the instantaneous wave‐free ratio (iFR) is also a useful tool to evaluate the coronary physiology. Recently, using a cut‐off value of 0.83 instead of 0.89 as a standard threshold was shown to increase the accuracy of coronary hemodynamics measurement in patients with severe AS and CAD.^19^ A more reliable examination of the ischemic burden for patients with severe AS and further risk/benefit assessment of the priorities of TAVI and PCI are needed.

Finally, the timing of PCI is a different issue. In their review, Cao et al. included the timing of PCI in a flowchart of the strategy of CAD management in TAVI patients.^20^ However, the recent article of Kumar et al. demonstrated in a pooled multi‐center registry that the adverse events rate was similar whenever PCI was performed for patients who received TAVI.^21^ There are many factors involved in this issue. For example, all components of the anatomy and the severity of coronary artery stenosis and aortic valve stenosis have to be taken into consideration. More evidence will be needed before reaching any definitive conclusion.

## 5 STUDY LIMITATIONS

This study has some limitations. It was a single‐center exploratory research and the sample size (especially patients with proximal LAD disease) was relatively small. The prognosis of the patients undergoing TAVI was relatively favorable, with 17 all‐cause deaths and 30 MACCEs during the follow‐up period. Further studies with a large sample size are warranted to validate the prognostic significance of patients with CAD after TAVI. Further research with large number of patients with proximal LAD disease is needed. However, the standardized TAVI procedure and the reliable follow‐up of the patients could help counteract these limits. In addition, because this was a retrospective analysis, there may have been selection bias. Although we minimized the risk of selection bias by performing a multivariate Cox regression analysis, there is a possibility of inconclusive results. There was also the limitation of defining CAD by visual assessments of CAG or CTA findings. PCIs guided by physiological assessments using FFR or iFR might have led to other results.

## 6 CONCLUSIONS

A lesion in the LM and a LAD proximal lesion are both a strong independent prognostic factor for patients with severe AS treated with a TAVI, and the performance of a PCI before the TAVI did not influence the present patients' mid‐term MACCEs or all‐cause mortality. For AS patients who undergo a TAVI and are complicated by CAD involving an LM or LAD proximal lesion, more intensive management may be necessary to improve clinical outcomes.

## CONFLICT OF INTEREST

The authors declared no potential conflict of interest.

## Supporting information

**Appendix S1**: Supporting information

## Data Availability

The datasets created during the study are available from the corresponding author on reasonable request
