# Supplementary material for: Influence of coronary artery disease and percutaneous coronary intervention on mid‐term outcomes in patients with aortic valve stenosis treated with transcatheter aortic valve implantation
Source: Clin Cardiol. 2021 May 25;44(8):1089–97. doi: 10.1002/clc.23655 (PMC8364726; doi:10.1002/clc.23655)

SUPPLEMENTAL MATERIAL

Influence of coronary artery disease and percutaneous coronary intervention on mid-term outcomes in patients with aortic valve stenosis treated with transcatheter aortic valve implantation

Toshiki Kaihara, MD, PhD[a], Takumi Higuma, MD, PhD[a]*, Masaki Izumo, MD, PhD[a], Nozomi Kotoku, MD, PhD[a], Tomomi Suzuki, MD, PhD[b], Haruka Kameshima, MD[a], Yukio Sato, MD, PhD[a], Shingo Kuwata, MD, PhD[a], Masashi Koga, MD[a], Takanobu Mitarai, MD[a], Mika Watanabe, MD[a], Kazuaki Okuyama, MD[a], Ryo Kamijima, MD, PhD[a], Yuki Ishibashi, MD, PhD[a], Kihei Yoneyama, MD, PhD[a], Yasuhiro Tanabe, MD, PhD[a], Tomoo Harada, MD, PhD[a], and Yoshihiro J. Akashi, MD, PhD[a]

[a]Division of Cardiology, Department of Internal Medicine, St. Marianna University School of Medicine, Kawasaki, Japan

[b]Department of Cardiology, St. Marianna University School of Medicine, Toyoko Hospital, Kawasaki, Japan.

***Address corresponding to:** Dr. Takumi Higuma, Division of Cardiology, Department of Internal Medicine, St. Marianna University School of Medicine, 2-16-1 Sugao, Miyamae, Kanagawa 216-8511, Japan. Tel.: +81-44-977-8111, Fax: +81-44-976-7093.

Email: higuma@marianna-u.ac.jp

List

- Supplementary Figure S1 in the supplement: Kaplan-Meier Curves in the CAD[LADp] group

Figure S1. Kaplan-Meier Curves in the CAD[LADp] group

Kaplan-Meier Curves for **(A)** MACCEs and **(B)** all-cause mortality for the patients performing PCI for LAD proximal lesion in the CAD[LADp] group (*blue*), and those not performing PCI for LAD proximal lesion (*green*).

CAD, coronary artery disease; LAD, left anterior descending artery; PCI, percutaneous coronary intervention; MACCEs, major adverse cardiovascular and cerebrovascular events.

Supple. Figure S1. Kaplan-Meier Curves in the CAD[LADp] group

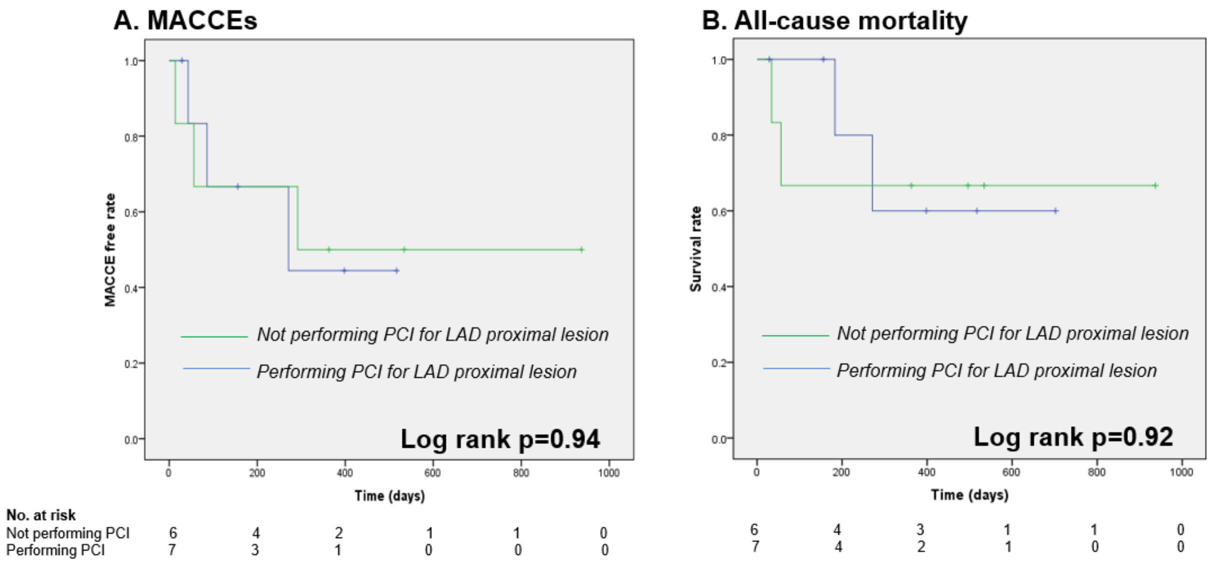

Supplement: Supplementary file 1 — Appendix S1: Supporting information [file CLC-44-1089-s001.pdf]
